# Nasal Associated Lymphoid Tissue of the Syrian Golden Hamster Expresses High Levels of PrP^C^

**DOI:** 10.1371/journal.pone.0117935

**Published:** 2015-02-02

**Authors:** Melissa D. Clouse, Ronald A. Shikiya, Jason C. Bartz, Anthony E. Kincaid

**Affiliations:** 1 Department of Biomedical Sciences, Creighton University, Omaha, Nebraska, United States of America; 2 Department of Medical Microbiology and Immunology, Creighton University, Omaha, Nebraska, United States of America; 3 Department of Pharmacy Sciences, Creighton University, Omaha, Nebraska, United States of America; University of Maryland School of Medicine, UNITED STATES

## Abstract

The key event in the pathogenesis of the transmissible spongiform encephalopathies is a template-dependent misfolding event where an infectious isoform of the prion protein (PrP^Sc^) comes into contact with native prion protein (PrP^C^) and changes its conformation to PrP^Sc^. In many extraneurally inoculated models of prion disease this PrP^C^ misfolding event occurs in lymphoid tissues prior to neuroinvasion. The primary objective of this study was to compare levels of total PrP^C^ in hamster lymphoid tissues involved in the early pathogenesis of prion disease. Lymphoid tissues were collected from golden Syrian hamsters and Western blot analysis was performed to quantify PrP^C^ levels. PrP^C^ immunohistochemistry (IHC) of paraffin embedded tissue sections was performed to identify PrP^C^ distribution in tissues of the lymphoreticular system. Nasal associated lymphoid tissue contained the highest amount of total PrP^C^ followed by Peyer’s patches, mesenteric and submandibular lymph nodes, and spleen. The relative levels of PrP^C^ expression in IHC processed tissue correlated strongly with the Western blot data, with high levels of PrP^C^ corresponding with a higher percentage of PrP^C^ positive B cell follicles. High levels of PrP^C^ in lymphoid tissues closely associated with the nasal cavity could contribute to the relative increased efficiency of the nasal route of entry of prions, compared to other routes of infection.

## Introduction

The normal isoform of the prion protein (PrP^C^) is a highly conserved mammalian glycophosphatidylinositol linked membrane protein expressed in tissues throughout the body [1]. PrP^C^ is found in highest concentrations in the central nervous system, but is also present in lower amounts in skeletal muscle, lung, intestine, autonomic ganglia, heart, and ovary [2, 3, 4]. Peripheral mucous associated lymphoid tissues, lymph nodes and spleen also express PrP^C^, where it has been localized to follicular dendritic cells (FDCs), intraepithelial lymphocytes and dendritic cells [3, 5]. While the highly-conserved nature and wide distribution of PrP^C^ suggest an important function, a definitive physiological role for PrP^C^ has not been determined and PrP^C^ null mice fail to display an overt phenotype [6].

Infectious prions consist of PrP^Sc^, a misfolded isoform of the host encoded PrP^C^, and are the causative agent of a class of progressive neurodegenerative diseases called the transmissible spongiform encephalopathies (TSEs) [7]. The TSEs include Creutzfeldt-Jakob disease in humans, scrapie in sheep and goats, bovine spongiform encephalopathy in cattle, chronic wasting disease in cervids, and transmissible mink encephalopathy in ranch raised mink. The TSEs have common characteristics that include extended incubation periods which can last years to decades, followed by development of clinical signs and a rapidly progressive disease course. PrP^C^ is required for prion infection as PrP^C^ knockout mice fail to replicate the agent and do not develop disease after inoculation with prions [8].

TSE diseases can be experimentally transmitted by a number of routes including intracerebral, per os, intranerve, intratongue, subcutaneous, and intraperitoneal routes of exposure [9, 10, 11, 12]. Inhalation of prion infected inoculum into the nasal cavity causes disease in hamsters, mice, sheep and deer [13, 14, 15, 16, 17]. Extraneural routes of inoculation are typically characterized by PrP^Sc^ accumulation in lymphoreticular system (LRS) tissues, particularly spleen, prior to neuroinvasion [18, 19]. Consistent with this feature, inhalation of inoculum by rodents results in early deposition of PrP^Sc^ in nasal associated lymphoid tissue (NALT), unencapsulated lymphoid tissue found directly inferior to nasal mucosa [13, 17]. This is of particular interest as inhalation of prions into the nasal cavity is 10–100 times more efficient compared to per os, considered to be the most common route of infection in natural prion disease [13, 15, 20].

The amount of PrP^C^ available for conversion is known to affect prion disease pathogenesis. Transgenic mice that produce one half the amount of PrP^C^ compared to wild type mice have longer incubation periods following intracerebral inoculation [21]. Aged mice express less PrP^C^ on FDCs compared to young mice and fail to show either clinical signs of prion infection or pathology as expected within their normal life span following intraperitoneal inoculation [22]. Taken together these observations suggest that the level of PrP^C^ available in LRS tissue has a measurable effect on the efficiency of prion infection. In this study we compared the abundance of PrP^C^ of selected lymphoid tissues collected from uninfected hamsters. We hypothesized that relatively high amounts of PrP^C^ in the NALT contribute to the increased efficiency of nasal cavity inoculations.

## Methods

### Ethics statement

This study was conducted in compliance with National Institutes of Health guidelines in the care and use of laboratory animals. All procedures involving animals were approved by the Creighton University Institutional Animal Care and Use Committee.

### Animals

Adult male Syrian golden hamsters (Harlan Sprague Dawley, Indianapolis, IN) were anaesthetized with isofluorane and killed via transcardial perfusion with phosphate buffered saline containing 5 mM ethylenediaminetetracetic acid (EDTA). Animals intended for immunohistochemistry (IHC) processing were subsequently perfusion fixed with periodate-lysine-paraformaldehyde (PLP) followed by immersion of the tissue in PLP for 5–24 hours at room temperature.

### Tissue collection

Lymphoid tissues including spleen (SP), Peyer’s patches (PP), submandibular lymph nodes (SLN) and mesenteric lymph nodes (MLN) were removed, placed into cassettes and stored in 70% ethanol at room temperature until processing. Heads (with jaw and tongue removed) were placed into decalcifying solution (Thermo Scientific, Kalamazoo, MI) for a total of two weeks at room temperature with a change of solution midway through the decalcifying process. Nasal cavities were blocked and embedded in paraffin as described previously [23]. Serial sections of each tissue were cut using a microtome at 7μm and collected on glass slides.

### Immunohistochemistry

Following deparafinization and rehydration endogenous peroxidases were blocked by immersion in 0.3% v/v hydrogen peroxide in methanol for 20 minutes. The slides were rinsed thoroughly with tris buffered saline containing 0.05% v/v Tween 20 (TTBS) and incubated for 30 minutes with 10% v/v normal horse serum in TTBS at room temperature. Slides were incubated at 4°C overnight with anti prion antibody 3F4 (2.6 μg/mL: Millipore, Temecula, CA) with 3% v/v normal horse serum in TTBS. Slides were rinsed with TTBS and incubated with biotinylated horse anti-mouse secondary antibody (1 μg/mL: Vector laboratories, Burlingame, CA) in 3% v/v normal horse serum TTBS for 30 minutes at room temperature. Signal amplification was performed using the Vectastain Elite ABC-HRP (Vector, Burlingame, CA) and diaminobenzidine reaction was used to visualize antigen location. The following controls were used to ensure specificity of IHC: use of a mouse IgG isotype control (Abcam, Cambridge, MA) in place of primary antibody and omission of either the primary or secondary antibodies with all other steps being the same. Lymphoid tissue sections not further than 140 μm apart were processed for PrP^C^ and examined using a Nikon Eclipse 80i light microscope. Images were captured with an Infinity 2 digital camera (Lumenera, Ottawa, ON) and ImageJ software (NIH, Bethesda, MD).

### Semi-quantitative calculation of PrP^C^ lymphoid follicles

The total number of lymphoid follicles and the number of lymphoid follicles expressing PrP^C^ from the SP, SLN, MLN, and PP from eight uninfected hamsters were examined. Percentages were calculated as the number of immunoreactive follicles divided by the total follicles per organ for each animal and further averaged per tissue type for the entire sample group.

### Tissue collection and preparation for Western blot

The SP, PP, SLN and MLN were collected, flash frozen and stored at -80°C. NALT collection technique was modified from a method previously described in mice [24]. Collection of NALT was accomplished by removing the jaw and muzzle anterior to the incisors. Tissues lateral and superior to the nasal cavity were trimmed without disturbing the nasal mucosa. The septal window was identified using a dissecting microscope and the septum was removed. The NALT, located deep to the mucosa on the floor of the nasal cavity, was removed, flash frozen and stored at -80°C. Lymphoid tissues were homogenized to 20% w/v in Dulbecco’s phosphate buffered saline, 1% v/v Triton X-100, 0.5mM EDTA and complete protease inhibitor (Roche Diagnostics, Mannheim, Germany). The homogenates were centrifuged for 30 seconds at 2000xg and the supernatant was removed and stored at -80°C. NALT samples were incubated with 2.5 U/μl benzonase (EMD Millipore, San Diego, CA) and 2mM magnesium chloride for thirty minutes on ice to decrease viscosity of the sample prior to Western blot procedures. Deglycosylation of select tissue samples was performed using PNGase F (New England Biolabs, Ipswich, MA) according to manufacturer protocol. Briefly, tissue homogenates were treated with 10% v/v 10x glycoprotein denaturing buffer at 100°C for ten minutes. The samples were incubated at 37°C with PNGase F (1 unit per 10 μg tissue) containing 10% v/v of G7 reaction buffer and 10% v/v NP-40 detergent.

### SDS-PAGE and Western blot

Sodium dodecyl sulfate polyacrylamide gel electrophoresis and Western blot procedures were performed as previously described [25]. Briefly, samples were size fractionated using NuPage 4–12% Bis-tris gels (Invitrogen, Carlsbad, CA) and transferred to polyvinylidene difluoride membrane (Millipore, Billerica, MA). After blocking the membranes with 5% w/v blotting grade blocker (BioRad, Hercules, CA) in TTBS at room temperature for a minimum of 30 minutes the membranes were probed using the mouse monoclonal anti-prion protein antibody 3F4 (0.2 μg/mL: Millipore, Temecula, CA) in 5% w/v Blotto/TTBS overnight at 4°C. Following washes in TTBS the membranes were incubated with peroxidase conjugated Affinipure donkey anti mouse secondary antibody (0.32 μg/mL: Jackson ImmunoResearch, West Grove, PA) in 5% w/v Blotto/TTBS for a minimum of one hour at room temperature. Following TTBS washes the membranes were developed with Super Signal West Femto (Pierce, Rockford, IL) and were imaged using a Kodak 4000R imager (Kodak, Rochester, NY). Quantification of PrP^C^ was performed using ImageQuant software (Kodak, Rochester, NY). Membranes were washed with TTBS then incubated with the anti β-actin mouse monoclonal antibody (0.005 μg/mL: Santa Cruz Biotechnology, Dallas, TX) for one hour at room temperature, washed with TTBS and exposed to peroxidase conjugated Affinipure donkey anti mouse secondary (0.32 μg/mL: Jackson ImmunoResearch, West Grove, PA) for one hour at room temperature. Washing, development and imaging of the membranes were performed as described above. β-actin protein abundance was used to normalize PrP^C^ protein levels between tissue samples. All lymphoid samples were examined in triplicate. A one-twentieth μg tissue equivalent of brain was examined relative to lymphoid tissue to ensure that the level of PrP^C^ was in the linear range of Western blot detection on all Western blots used for quantification. Normalized PrP^C^ abundance values were calculated as a percentage of the uninfected brain PrP^C^ intensity average in order to standardize lymphoid PrP^C^ intensity measurements between Western blots. PrP^C^ migration patterns were compared by plotting intensity of PrP^C^ signal against migration distance allowing graphic visualization of the relative molecular weight populations of PrP^C^ present in each sample.

### Statistical analysis

PrP^C^ abundance was compared using one way analysis of variance (ANOVA); significance value was set at P<0.05. Post hoc testing was performed using Tukey’s honestly significant difference (HSD) test. ANOVA and Tukey’s HSD tests were both performed using Graphpad Prism software V6.0 (San Diego, CA).

## Results

### The abundance of total PrP^C^ in NALT homogenates was greater compared to the other lymphoid tissues examined

**Fig 1:**
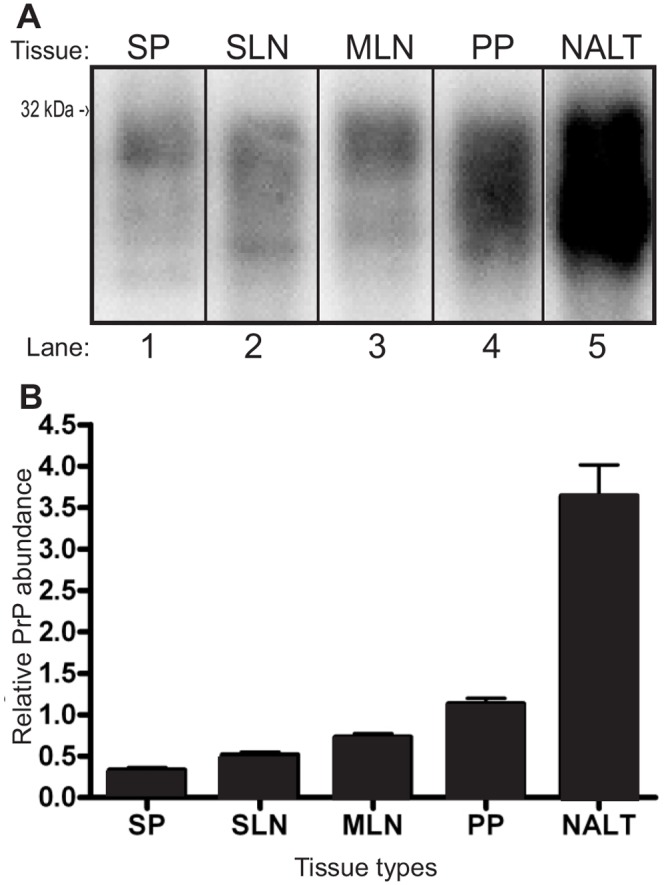
NALT contains significantly more PrP^C^ than other lymphoid tissues. A) Western blot analysis and B) normalized quantification of lymphatic tissue PrP^C^ abundance. SP—spleen, SLN—submandibular lymph node, MLN—mesenteric lymph node, PP—Peyer’s patch, NALT—nasal associated lymphoid tissue.

NALT contained approximately 3, 5, and 6 fold the amount of PrP^C^ per μg equivalent of tissue compared to PP, MLN and SLN respectively (Fig. 1B). SP contained the lowest amount of PrP^C^ per μg equivalent of the examined tissues (Fig. 1B). Analysis of the PrP^C^ abundance with ANOVA testing indicated that lymphoid tissues from different organs contain significantly (p<.05) different amounts of PrP^C^. All one on one comparisons of PrP^C^ from lymphoid tissues performed using Tukey’s honestly significant difference post hoc testing were significantly (p<0.05) different with the exception of SP versus SLN and MLN versus SLN.

### Distinctive PrP^C^ migration patterns were observed between tissue types

**Fig 2:**
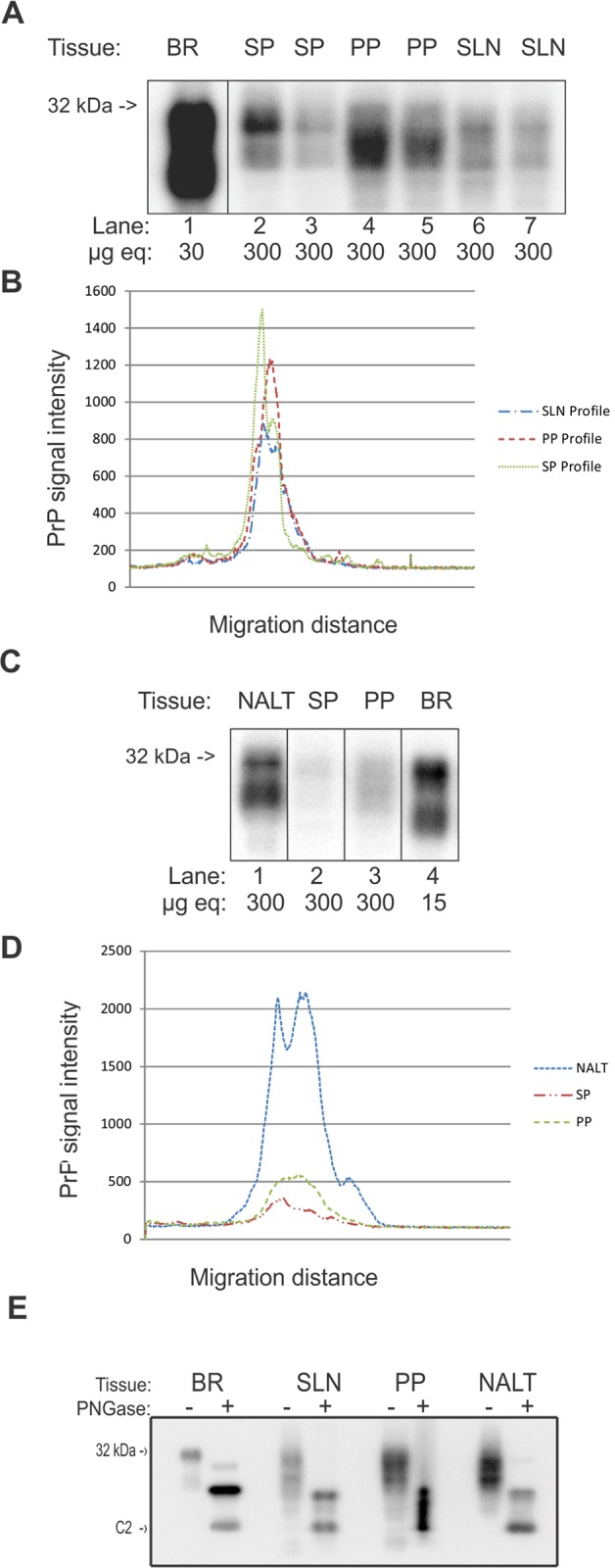
PrP^C^ migration patterns were distinct for different lymphoid tissues. A) Migration banding patterns are consistent when comparing individual animal homogenates. SP, SLN and PP homogenates from individual animals migrate similarly. B) Line graph of lane intensity analysis demonstrating peak differences in intensity of PrP^C^ migration. C) Western blot of lymphoid tissue (NALT, SP, and PP) and BR control. D) Line graph of lane analysis of PrP^C^ intensity of lymphoid tissue indicates intensity difference and migration profile of NALT. E) Comparison of PNGase treated and untreated PrP^C^ from lymphoid tissue (SLN, PP and NALT and BR) demonstrates variable levels of full length and truncated PrP^C^ in samples. BR—brain, SP—spleen, PP—Peyer’s patch, SLN—submandibular lymph node, NALT—nasal associated lymphoid tissue.

PrP^C^ from PP contained a mixed molecular weight population with no obvious prevalent group while SP and SLN PrP^C^ contained two distinct migration patterns (Fig. 2A lanes 4–5, 2–3, and 6–7). The abundance of PrP^C^ from SLN was equally distributed between relatively higher and lower molecular weights while PrP^C^ from the SP was mainly comprised of a higher molecular weight population (Fig. 2B). PrP^C^ from NALT exhibited a unique migration pattern compared to the other examined lymphoid tissues with two distinct bands but a prevalence of the lower weight population (Fig. 2C). PNGase treatment of SLN, PP and NALT indicated differences in overall ratios of full length PrP^C^ to truncated forms (Fig. 2E). While NALT and SLN were both composed of two predominant forms of PrP^C^, PP total PrP^C^ consisted of multiple truncated forms of PrP^C^ which were between the molecular weights of full length and the truncated fragment C2.

### PrP^C^ was localized to B cell follicles in lymphoid tissue

**Fig 3:**
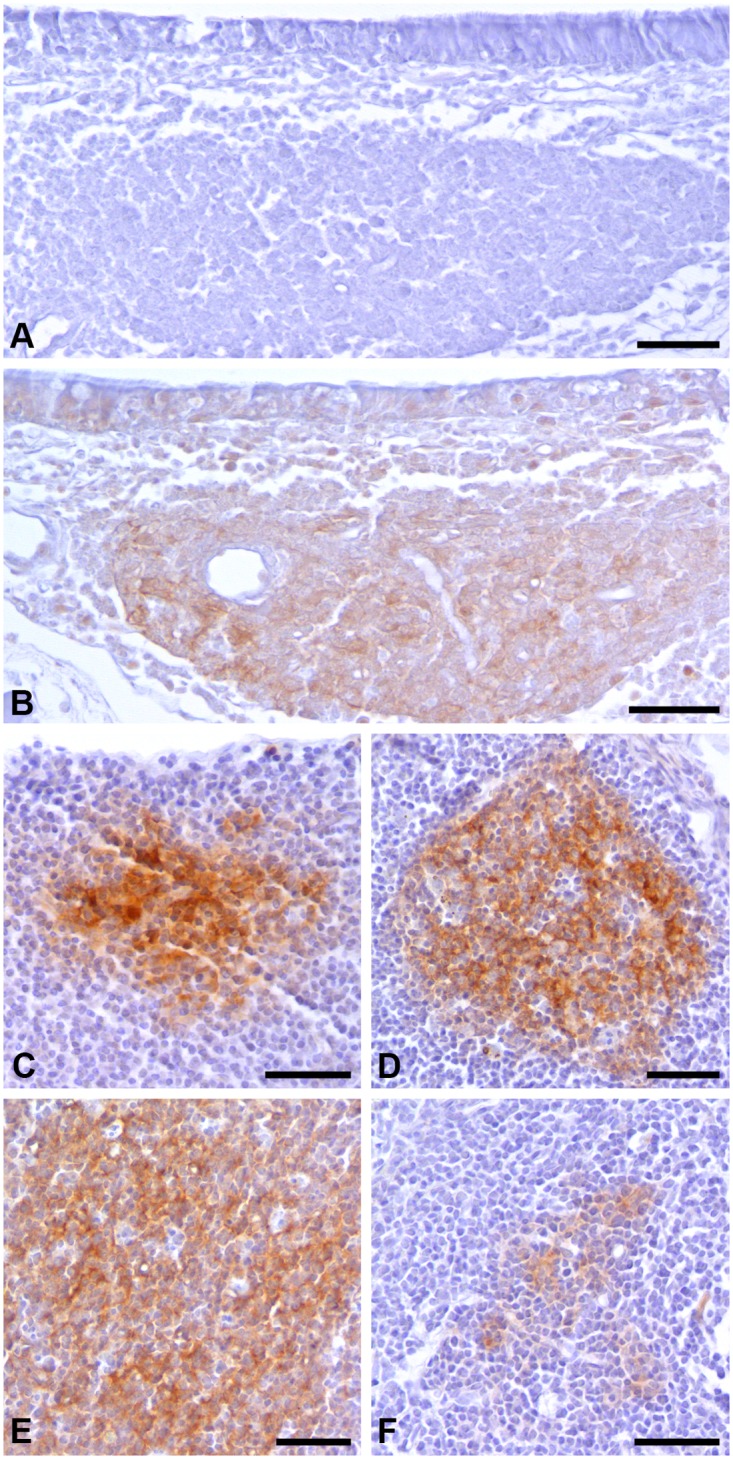
Immunohistochemistry (IHC) of lymphoid tissues demonstrating presence and localization of PrP^C^ within B cell follicles. IHC was performed on A/B) NALT, C) MLN, D) SLN, E) PP and F) SP with the anti-PrP antibody 3F4 (B-F) or an isotype control (A). The tissue sections were processed identically using the same reagents at the same time to illustrate relative differences in PrP^C^ expression between tissues. The scale bar represents 100 μm. NALT—nasal associated lymphoid tissue, MLN—mesenteric lymph node, SLN—submandibular lymph node, PP—Peyer’s patch, SP—spleen.

PrP^C^ immunoreactivity was visualized as a brown, diffuse reaction product between lymphocytes. PrP^C^ immunoreactivity was mainly observed in B cell follicles of examined lymphoid tissues (Fig. 3B-F), however, a distinct difference in the percentage of PrP^C^ positive follicles in the different lymphoid tissues was noted. The ratio of PrP^C^ containing follicles was greatest in PP (89.7%±12.5%) with smaller percentages of PrP^C^ positive follicles identified in MLN and SLN (55.2%±17.3% and 67.6%±14.2%) and the smallest ratio of PrP^C^ positive follicles in SP (20.9%±7.7%). NALT was characterized by the widespread distribution of PrP^C^ immunoreactivity through the entire structure (Fig. 3B). For all of the tissues PrP^C^ immunoreactivity was consistent with the morphology and location of FDCs [26]. Omission of primary or secondary antibodies, or use of the isotype control resulted in a complete lack of staining (Fig. 3A).

## Discussion

The primary finding of this work is that NALT contains a relatively greater amount of total PrP^C^ compared to other lymphoid tissues and that lymphoid tissues express distinct levels of PrP^C^ as demonstrated by both Western blot and IHC. The importance of PrP^C^ abundance is apparent in an *in vitro* model of prion infection. Protein misfolding cyclic amplification (PMCA) using brain homogenate from mice overexpressing PrP^C^ as substrate resulted in more efficient conversion of PrP^Sc^ than substrate from wild type mice [27]. This finding indicates the level of available PrP^C^ is a contributing factor in the conversion efficiency of endogenous PrP^C^ to pathological PrP^Sc^. This effect is also seen in *in vivo* models as mice engineered to express decreased levels of PrP^C^ have longer incubation periods following inoculation with mouse adapted prion disease than wild type control mice [21, 28].

The relative differences in total PrP^C^ amounts between lymphoid tissues positively correlates with the PrP^Sc^ levels in prion-infected animals. Hamsters peripherally inoculated with the hyper strain of hamster adapted transmissible mink encephalopathy (TME) demonstrate higher amounts of PrP^Sc^ in lymph nodes than spleen at clinical stage of disease [29]. This does not appear to be unique to hamsters as lymph nodes from mink subcutaneously infected with TME are infectious several months before spleen and contain higher titers of infectious agent once the clinical stage of disease has been reached [30]. Peyer’s patches and lymph nodes from terminal mice orally infected with both scrapie and bovine spongiform encephalopathy also consistently contain higher levels of PrP^Sc^ than spleen [31].

Differences in PrP^C^ migration patterns that were observed between lymphoid tissues in hamsters may be due to a difference in glycoform ratios in the tissue homogenates. The presence of PrP^C^ with distinctive ratios of glycoforms has been previously observed in transgenic mice that selectively expressed tissue-specific PrP^C^, which is consistent with our observation that different tissues possessed distinct patterns of glycosylation [35]. Glycosylation of PrP^C^ can affect the efficiency of PrP^Sc^ formation. The effect of PrP^C^ glycoforms on conversion to PrP^Sc^ can be influenced by the strain and species of PrP^Sc^. For example, in hamsters, diglycosylated PrP^C^ supports conversion of Sc237 but in mice the prion strain RML requires the presence of unglycosylated PrP^C^ for efficient conversion [32]. Glycosylation may affect the efficiency of PrP^Sc^ formation by making the conformation of the cellular protein more similar to that of the disease associated protein. PrP^C^ produced by cells engineered to hinder post translational modifications of the protein, including glycosylation, has biochemical properties more commonly associated with PrP^Sc^ such as resistance to protease digestion [33] and similar treatment of other glycoproteins increases their insolubility in detergents [34].

Tissue specific populations of full length and truncated forms of PrP^C^ may contribute to the difference in PrP^C^ migration patterns observed between lymphoid tissues. β-cleavage of PrP^C^ occurs under normal physiological conditions resulting in the C2 truncated PrP^C^ fragment [35]. This cleavage event can vary between tissues, leading to primary structural differences in total populations of PrP^C^ [36]. Interestingly, deglycosylated PrP^C^ from PP appears to consist of multiple truncated forms, suggesting distinctive β-cleavage patterns on that tissue. This is in agreement with a recent *in vitro* study in which enzymatically driven β-cleavage was observed at multiple sites within the octarepeat sequence of PrP^C^ [37], however, we cannot exclude the possibility that the multiple forms of PP PrP^C^ occur *ex vivo*. Our results are consistent with a study which revealed multiple species of PrP^C^ defined by differences in post translational modifications, namely glycophosphotidylinositol moieties and glycol groups, and in primary structure of the protein itself [38]. Given the complexity of PrP^C^ structure, additional characterization of NALT PrP^C^ structure may increase our understanding of unique aspects of the nasal route of inoculation. Overall, the increased abundance of PrP^C^ in the NALT may contribute to the increased efficiency of extranasal prion infection compared to the *per os* route.
